# Germline ancestry influences the evolutionary disease course in lung adenocarcinomas

**DOI:** 10.1111/eva.12964

**Published:** 2020-04-17

**Authors:** Alina Schenk, Saioa López, Maik Kschischo, Nicholas McGranahan

**Affiliations:** ^1^ Department of Mathematics and Technology University of Applied Sciences Koblenz Remagen Germany; ^2^ Cancer Genome Evolution Research Group University College London Cancer Institute London UK; ^3^ Cancer Research UK Lung Cancer Centre of Excellence University College London Cancer Institute London UK

**Keywords:** cancer, genomics, germline ancestry, somatic evolution

## Abstract

Precision medicine relies on targeting specific somatic alterations present in a patient's tumor. However, the extent to which germline ancestry may influence the somatic burden of disease has received little attention. We estimated the genetic ancestry of non‐small‐cell lung cancer (NSCLC) patients and performed an in‐depth analysis of the influence of genetic ancestry on the evolutionary disease course. Compared with European Americans (EA), African Americans (AA) with lung adenocarcinoma (LUAD) were found to be significantly younger and smoke significantly less. However, LUADs from AAs exhibited a significantly higher somatic mutation burden, with a more pronounced tobacco carcinogen footprint and increased frequencies of alterations affecting cancer genes. Conversely, no significant differences were observed between lung squamous cell carcinomas (LUSC) from EAs and AAs. Our results suggest germline ancestry influences the somatic evolution of LUAD but not LUSC.

## 1 INTRODUCTION

Large‐scale sequencing projects, including the Cancer Genome Atlas (TCGA) and the International Cancer Genome Consortium (ICGC), have revolutionized our understanding of the genomic basis of cancer. Studies building upon these data have identified scores of cancer‐associated genes (Bailey et al., 2018; Lawrence et al., 2014) and revealed many of the mutational processes underpinning cancer development (Alexandrov et al., 2013, Alexandrov et al., 2015, Alexandrov et al., 2020).

However, to date most studies have not focused on the potential influence of germline ancestry on cancer development and cancer evolution. Emerging data suggest germline ancestry can influence disparities in cancer care and the subsequent disease course. For instance, women with African ancestry (AA) have been reported to have higher breast cancer mortality, compared to women with European ancestry (EA), which has been associated with a higher occurrence of the more aggressive triple‐negative form (Daly & Olopade, 2015)**.** Colorectal cancer has been shown to be more lethal in both AA men and women relative to EA individuals (O'Keefe et al., 2015). These are not isolated observations as AA individuals have unfavorable health outcomes within numerous cancer types (Polite et al., 2017). The TCGA PanCan Atlas Germline Working Group also reported that on average, AA individuals harbor more germline predisposing variants relative to EA (Huang et al., 2018), and an enrichment of *TP53* mutations (Yuan et al., 2018).

Here, we interrogate differences in the cancer genomes of EA and AA individuals for two subtypes of non‐small‐cell lung cancer, lung adenocarcinoma (LUAD), and lung squamous cell carcinoma (LUSC) using data from TCGA (Campbell et al., 2016). We explore differences in the age of diagnosis and in the disease progression between both ethnic ancestries. We also investigate differences in the strength of association between tobacco exposure and lung cancer development.

## 2 METHODS

### 2.1 Defining germline ancestry

**Figure 1:**
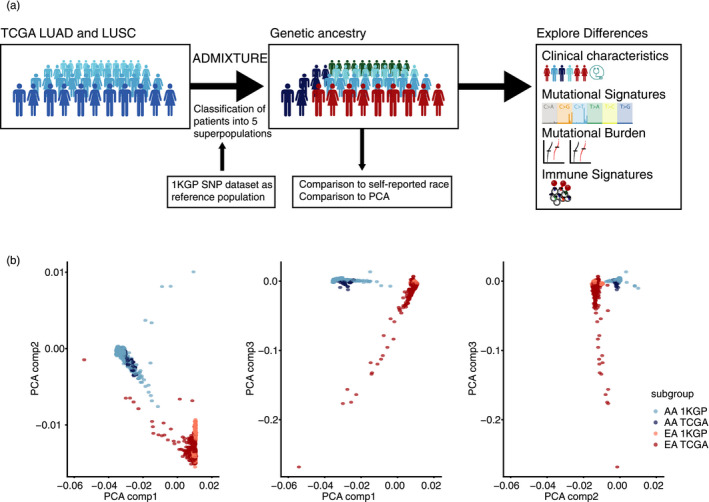
(a) Overview of the workflow. Ancestry estimation was performed by applying ADMIXTURE to the TCGA LUAD and LUSC cohort using 1KGP germline data as reference population and setting *k* = 5, matching the number of super‐populations in the 1KGP cohort. Using a threshold of 0.8, classification of TCGA data was performed. Independently, a principal component analysis (PCA) was applied to the TCGA and 1KGP germline data to evaluate the consistency of the ADMIXTURE analysis. Subsequently, a comparison of the EA and AA cohort was conducted. (b) Scatter plot of PCA component 1 against PCA component 2, PCA component 1 against PCA component 3 and PCA component 2 against PCA component 3 for 1KGP (light red for EA and light blue for AA) and for TCGA (dark red for EA and dark blue for AA). Reassuringly, the estimated ancestries in TCGA and given ancestries in 1KGP in each group cluster together very well without any overlap to the other group. An equivalent plot for LUSC is shown Figure S1

The genetic ancestries of the TCGA LUAD and LUSC cohorts were determined using reference populations from the 1,000 Genomes Project (1KGP) (Liu et al., 2013) and applying ADMIXTURE (Liu et al., 2013). The workflow is illustrated in Figure 1.

ADMIXTURE estimates ancestries in a model‐based manner from large autosomal SNP genotype datasets with maximum likelihood estimation applying a block relaxation approach which is a fast numerical optimization algorithm (Liu et al., 2013). It models the probability of observed genotypes using ancestry proportions and population allele frequencies, simultaneously estimating population allele frequencies along with ancestry proportions. The supervised approach used in this analysis requires a training dataset as well as the number of clusters to be estimated. In this case, the number of clusters was set to *k* = 5, matching the number of different super‐populations in the 1KGP (EA (European American), AA (African American), SAS (South Asian), EAS (East Asian), and AMR (American)).

For each TCGA sample in the LUSC and LUAD cohort, a patient was considered as EA or AA, when the proportion for the European or African cluster was higher than 0.8, respectively, resulting in 36 AA and 448 EA patients in LUAD and 19 AA and 450 EA patients in LUSC. The results did not change qualitatively if the threshold was varied between 0.7 and 0.9. American, South Asian, and East Asian individuals were defined in the same way. The estimated ancestry was compared to the race reported in the TCGA clinical data to cross‐check the results of the ADMIXTURE run.

After defining the two groups, clinical features, more precisely age at initial diagnosis, lifetime tobacco exposure measured by pack‐years (number of cigarettes smoked per day multiplied by the number of years smoked divided by 20) as well as tumor stage, were compared using *t* test, Wilcoxon test, and Fisher's exact test.

### 2.2 Exploring driver differences

For the list of cancer genes (Bailey et al., 2018; Lawrence et al., 2014; Martincorena et al., 2017) (Table S1 for LUAD and Table S2 for LUSC), the frequency of patients having at least one nonsilent mutation in cancer genes was determined for each group and each gene and was compared by using Fisher's exact test. False discovery rate (FDR) control was used to account for multiple testing. The statistical significance of relative frequency estimates was indicated by 95% confidence intervals.

### 2.3 Mutational signatures

Mutational signatures within the LUAD and LUSC cohort were detected by applying a Bayesian variant of the non‐negative matrix factorization (NMF) algorithm described in (Kim et al., 2016). K* detected contexts were compared and matched to already published COSMIC Signatures using cosine similarity. Here, K* indicates the optimal number of contexts given by the algorithm. NMF estimates two matrices W and H representing mutational signatures and their occurrence patterns in each patient. After estimating the matrices W and H from the algorithm for each found context, the cohort was separated into EA and AA and the activity of the signatures among the two groups was compared implementing a Wilcoxon test. Linear regression was used to check for associations of signatures with clinical features in each group. Propensity score matching was used to control for potential confounders.

### 2.4 HLA LOH and immune deconvolution

The LOH (loss of heterozygosity) status for the TCGA cohort was collected by running LOHHLA, a computational tool to determine HLA allele‐specific copy number from sequencing data (McGranahan et al., 2017). LOH can occur in different ways: Either all class I HLA alleles A, B, and C are lost, any of these alleles is lost, or none is lost. After assigning LOH status for each patient, we used Fisher's exact test to compare whether all or any of the three mentioned HLA alleles are lost or whether none of them is lost.

Immune signatures as described by (Danaher et al., 2017) were used to compare immune scores among EA and AA individuals. Danaher suggests to calculate immune scores as follows:

Assuming each marker gene for a certain cell type j is present at a fixed but unknown number of cells $c_{j}$ , the average log‐transformed expression of the marker genes in that cell type is equal to the log‐transformed abundance of the cell type, plus an unknown constant. Let $x_{\mathrm{ij}}$ be the expression value of marker gene i and let $n_{j}$ be the number of marker genes in cell type j , the cell type score for cell type j can be obtained as follows:

$$\log\log\left(c_{j}\right)=\frac{1}{n_{j}}\sum_{i=1}^{n_{j}}\log\log\left(x_{\mathrm{ij}}\right)+\theta_{j}$$

$\theta_{j}$ is an unknown constant (Danaher et al., 2017).

## 3 RESULTS

### 3.1 Estimating the genetic ancestries in the TCGA LUAD and LUSC cohort

To explore the underlying germline ancestry of each and every TCGA patient, ADMIXTURE was applied (Liu et al., 2013), using the 1KGP for training data (Genomes Project et al., 2015) (Figure 1). The supervised approach used in this analysis requires a training dataset as well as the number of clusters to be estimated. In this case, the number of clusters was set to *k* = 5, matching the number of different superpopulations in the 1KGP (EA (European American), AA (African American), SAS (South Asian), EAS (East Asian), and AMR (American)). Each patient could then be assigned a specific superpopulation (using a threshold of 0.8). All patients that could not clearly be allocated to a specific ancestry (using a threshold of 0.8) were classified as “other.” EA and AA were the predominant groups (LUAD: 448 EA and 36 AA; LUSC: 450 EA and 19 AA). There was no clear difference in the separation of the patient cohort into the different subpopulation between the two lung cancer types (*p* = .082, Fisher's exact test).

**Figure 2:**
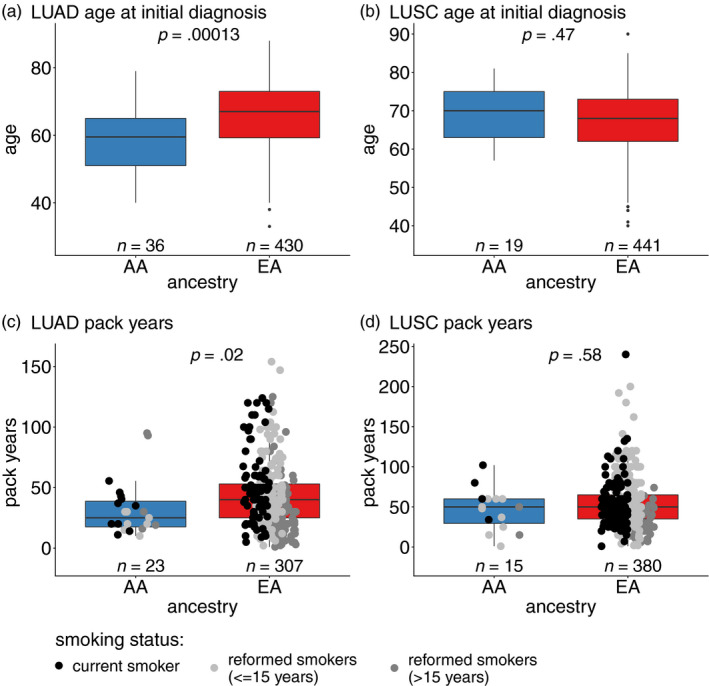
Comparison of age at initial diagnosis in LUAD (a) and LUSC (b) and tobacco exposure measured by pack‐years for LUAD (c) and LUSC (d). AA patients seem to be younger when diagnosed with cancer compared to EA individuals in LUAD (*t* test, *p* = .00013). Furthermore, EA patients with LUAD have on average a higher tobacco exposure (Wilcoxon test, *p* = .02). The results are similar after splitting the cohort in current and reformed smokers For LUSC, there were no significant results

To confirm the results of the ADMIXTURE, a principal component analysis (PCA) of the TCGA and 1KGP germline data was applied. Reassuringly, the analysis revealed strong concordance between samples grouped by ancestral genotype (Figure 2 and Figure S1).

### 3.2 AAs with LUAD exhibit different clinical features compared to EA

**Figure 3:**
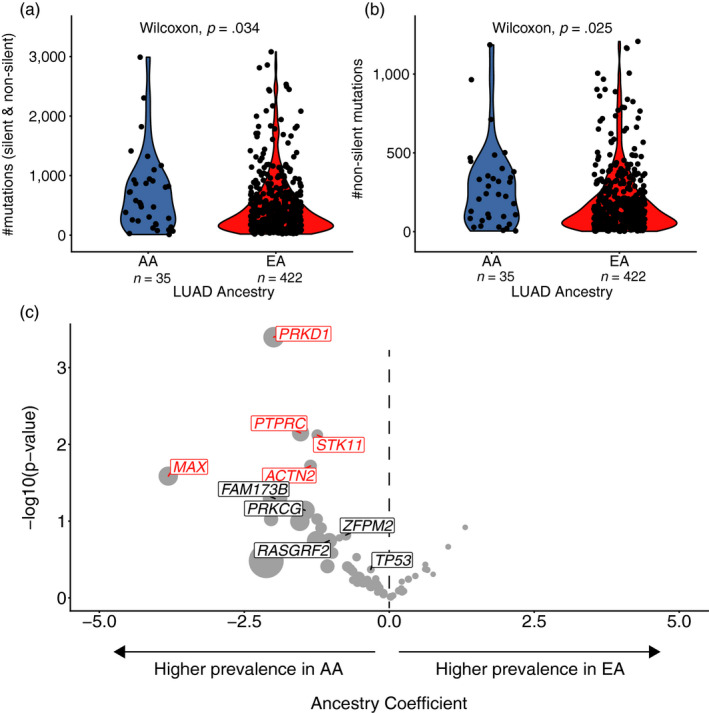
(a) Number of mutations for each patient for each group shown and compared in a boxplot. (b) shows the number of nonsilent mutations per group compared in a boxplot. Increased mutation burden remains significant when restricting the analysis to nonsilent mutations. (c) shows the results of logistic regressions for each cancer gene. Genes with a negative coefficient are enriched in AA, while those with a positive coefficient are enriched in EA. Genes that remained significant after accounting for mutation burden and age at initial diagnosis are highlighted in red, genes revealed by Fisher's exact test significance are colored black. Dot sizes indicate the ratio of relative frequencies (AA frequency/EA frequency)

Intriguingly, clinical features such as age at initial diagnosis and pack‐years were significantly different among AA and EA patients with LUAD, but not in LUSC (Figure 2). EA patients with LUAD were older at diagnosis (*p* = .00013, *t* test for age), and, on average, they also smoked less than EA individuals (*p* = .02, Wilcoxon test for pack‐years). Stratifying the groups by gender revealed the same results regarding age—the AA male and female patients in LUAD were typically older than EA male and female patients, whereas no difference in LUSC was detectable (Figure 3).

To account for a possible relationship between age and pack‐years in LUAD, we used a linear model and found no evidence for such an association (*p* = .22, effect size = 0.191). Dividing the patients into groups according to smoking history as shown in Figure 3, the same results were observed. Here, p‐values and significance levels should be treated with caution since due to the small number of patients within subgroups power of statistical testing is diminished. Linear regression models of age against ancestry and pack‐years against ancestry support the hypothesis of EA being older at their age of diagnosis in LUAD and of EA having a higher tobacco exposure than AA. Interestingly, no significant associations in LUSC for age nor pack‐years were observed.

### 3.3 Differences in mutation burden and selection of certain mutations may lead to distinct evolutionary tumor progress

Given the younger age and lower smoking exposure in AA compared to EA LUAD patients, we next asked whether we could observe differences in the somatic landscape of LUAD tumors between these two groups.

**Figure 4:**
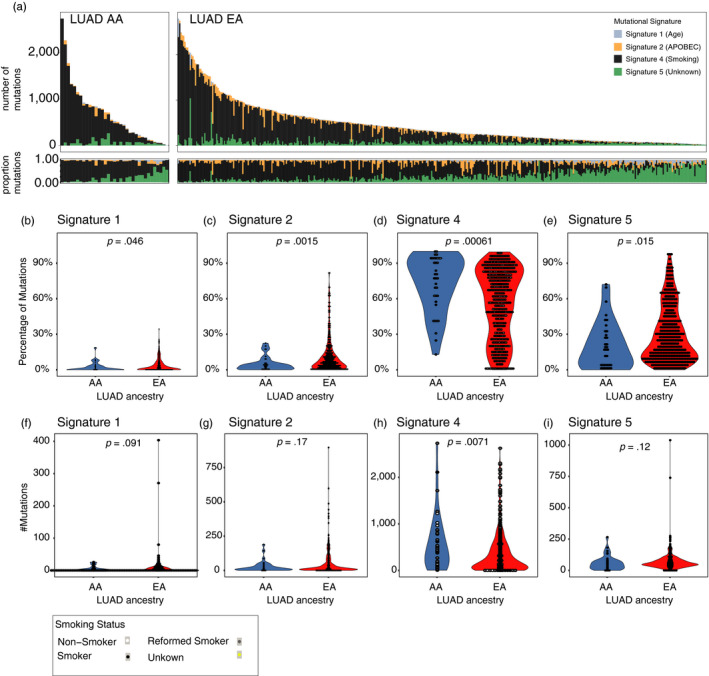
Results of mutational signature analysis. (a) shows the number of mutations contributing to the four signatures (CS1, CS2, CS4 and CS5) for each patient separately for EA and AA. (b), (c), (d), and (e) show the comparison of CS1 (b), CS2 (c), CS4 (d), and CS5 (e) activity in both ancestries. (f), (g), (h), and (i) show the comparison of mutations contributing to the respective signature in both ancestries. We see differences in activities in all four signatures. In CS4 (smoking signature) (d, h) AA individuals harbored a significantly higher burden of CS4 mutations although smoking less. As shown in (c), CS2, the APOBEC signature, was found to be significantly lower in the AA cohort. (b, f) and (e, i) visualize the EA group having a higher contribution to the aging signatures CS1 and CS5 as it was to be expected. After applying propensity score matching (PSM) considering age and tobacco exposure in CS4, the difference completely disappears. When applying PSM to CS2 considering age and APOBEC3B expression, the difference still remains after PSM

Strikingly, despite smoking less and being significantly younger, AA LUAD tumors harbored a significantly higher number of nonsilent mutations compared to their EA counterparts (Wilcoxon test, *p* = .025). Conversely, no significant differences were observed for LUSC tumors (Figure S2). On average, AA LUAD tumors exhibited a mutation burden of 279 (minimum = 4, 1st quantile = 86.5, median = 236, mean = 279.143, 3rd quantile = 367, maximum = 1,185, standard deviation = 264.9057), equating to 4.961 mutations per year, while EA LUAD tumors the average mutation burden was 190 (minimum = 2, 1st quantile = 49, median = 124, mean = 190.877, 3rd quantile = 251, maximum = 1,207, standard deviation = 209.1102), equating to only 3.084 mutations per year (Figure 4).

Consistent with a higher mutation burden in AA LUAD, we also observed that the relative frequency of mutations in established cancer genes was higher in this cohort. Fisher's exact test per cancer gene revealed *ACTN2, TP53, ZFPM2, STK11, RASGRF2, PRKCG, PRKD1, PTPRC, FAM173B* to occur at significantly different frequency between the groups (results before FDR correction). On average, AA have significantly more mutated cancer genes per patient than EA (Wilcoxon test, *p* = .002531, EA: median = 3, mean = 3.643; AA: median = 5, mean = 5.5). Because this could be a result of AA individuals having in general a higher mutation burden, logistic regression models were performed, considering the individual mutation burden, age at initial diagnosis, and ancestry in each model. Five cancer genes mutated at greater frequency in AA LUAD tumors (*MAX*, *ACTN2*, *PRKD1, PTPRC*, *STK11*) remained significant after accounting for mutation burden and age at diagnosis (Figure 3).

### 3.4 Differences in mutational signatures between EA and AA

To investigate the mutational processes underpinning the increased burden in LUAD patients, we applied Bayesian non‐negative matrix factorization (NMF) to the LUAD cohort (Kim et al., 2016). In total, four mutational signatures were identified (Figure 4a), which corresponded to previously identified signatures. The four signatures identified could be linked to COSMIC Signature 4 (CS4, cosine similarity 0.96), a signature linked to tobacco exposure, CS1 (cosine similarity 0.911), associated with spontaneous deamination of methylated cytosines and thought to correlate with patient age, CS2 (cosine similarity 0.854) linked to APOBEC‐mediated mutagenesis and CS5 (cosine similarity 0.899), whose etiology is unknown, but has been found to correlate with patient age (Alexandrov et al., 2015, Alexandrov et., 2020, Alexandrov et., 2013).

As expected, we observed a significantly higher contribution of CS1 and CS5 mutations in EA compared to AA LUADs, consistent with the older age of EA patients. However, strikingly, although AA patients smoked less, we identified that their tumors harbored a significantly higher burden of CS4 mutations. Conversely, CS2, which has been linked to APOBEC‐mediated mutagenesis, was found to be significantly lower in the AA cohort, with no patients exhibiting a dominant APOBEC signature (>25% of mutations) (Figure 4).

To evaluate whether these differences are influenced by patient age, we applied a 3:1 propensity score matching, meaning that one AA individual was matched to three EA individuals. Interestingly, when adjusting for pack‐years, the difference in CS4 mutations still remains whereas when adjusting for age, the difference almost disappears completely. Also, when considering both, age and pack‐years for the calculation of the propensity scores, the difference almost disappears completely (Figure 4). This suggests that conceivably the differences between AA and EA observed in mutational signatures may be linked to patient age and the fact that AA LUAD patients tend to be significantly younger than EA LUAD patients. However, when we applied the same procedure to CS2, calculating propensity scores considering age and APOBEC3B expression, the significant difference remains (Figure 4) (No clear differences in mutational signatures were observed between AA and EA LUSC tumors, Figure S3).

Taken together, these data suggest that the differences in mutational burden between AA and EA LUADs may reflect differences in the impact of mutational processes sculpting the cancer genome. However, potentially some these differences may be driven by differences in patient age.

### 3.5 Immune infiltration is different among EA and AA in LUAD

Finally, given the higher burden of mutations, and thereby also potentially neoantigens (Rooney, Shukla, Wu, Getz, & Hacohen, 2015) in AA LUAD tumors, we considered whether AA show differences in the loss of heterozygosity in the HLA‐class I alleles, a potential mechanism of immune escape (McGranahan et al., 2017; Rosenthal et al., 2019).

**Figure 5:**
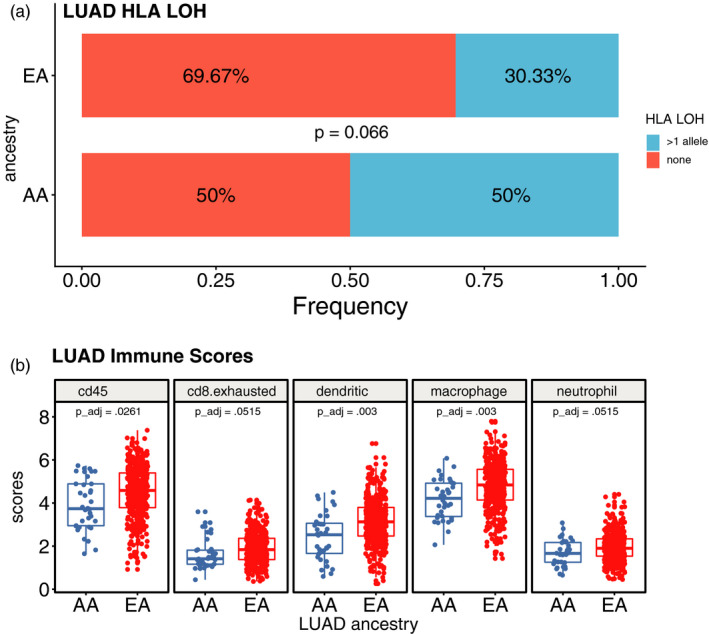
Comparison of HLALOH (a) and immune scores (b). The percentage of patients having any or all HLA alleles lost is higher when compared to the EA group. One out of two patients has at least one HLA allele lost. Five immune scores remained significant after applying FDR‐control. In all cases shown here, EA individuals show a higher immune score

Comparison of HLA LOH revealed that AA LUADs were more likely to exhibit HLA LOH (Figure 5), although this was not statistically significant (*p* = .066). Conversely, EA LUAD tumors were found to exhibit more signatures of immune infiltration, with five out of 16 immune signatures measured found to be lower in AA compared to EA LUADs.

## 4 DISCUSSION

We used germline and somatic sequencing data from TCGA to investigate the influence of germline ancestry on the somatic evolution of NSCLCs. Each NSCLC patient was found to be one of five superpopulations (AA, EA, SAS, EAS, and AMR), with EA (448 LUAD and 450 LUSC) and AA being the major groups (36 LUAD and 19 LUSC).

Intriguingly, we observed clear clinical differences between EA and AA LUAD, but not LUSC patients. EA LUAD patients were found to be significantly older and exhibited significantly distinct smoking history, with a significantly higher average pack‐year. These results are in keeping with significant differences in smoking history reported by (Campbell et al., 2017).

Interestingly, despite being significantly younger and smoking significantly less, on average, AA LUADs harbored a significantly elevated mutation burden compared to their EA counterparts. This remained significant when restricting the analysis to nonsilent mutations. Focusing specifically on driver alterations, a subset of genes were found to be more likely to be mutated in AA compared to EA LUADs. This suggests that germline ancestry may influence the selection for subsequent somatic alterations. However, consistent with previous studies, no clear differences were observed with regard to targetable alterations (Campbell et al., 2017). Nevertheless, it is worth considering that the higher burden of mutations in AA LUADs may render these tumors particularly susceptible to immune checkpoint blockade (Rizvi et al., 2015).

Consistent with previous reports, we observed four distinct mutational signatures within TCGA LUAD tumors (Campbell et al., 2016), including those related to smoke exposure (CS4), aberrant APOBEC activity (CS2), and aging (CS1 and CS5). In keeping with their older age, EA LUAD tumors were associated with an elevated burden of aging‐related mutations. Strikingly, AA tumors exhibited a greater preponderance of CS4—smoking‐related—mutations. These data imply that despite being associated with less tobacco exposure AA tumors may be more susceptible to the mutagenic insults of tobacco smoke. Conceivably, this may suggest underlying germline susceptibility which reduces the repair of bulky DNA adducts linked to carcinogens in cigarettes. It is notable that no significant difference was observed in LUSC tumors. Thus, these data suggest germline ancestry may specifically influence the LUAD tumor evolutionary trajectories.

Future studies, including larger AA LUAD cohorts, will be required to validate these findings and to obtain a deeper understanding of how germline ancestry impacts upon detoxification of mutagenic insults. Moreover, further investigation into other genomic factors such as mutations in noncoding regions, epigenetic alterations, and gene expression changes as well as socioeconomic variables beyond smoking behavior and access to health care may be required to fully explain these disparities.

## CONFLICT OF INTEREST

None declared.

## Supporting information

Fig S1

Fig S2

Fig S3

Fig S4

Table S1‐S2

## Data Availability

The results published here are in part based upon data generated by The Cancer Genome Atlas pilot project established by the NCI and the National Human Genome Research Institute. The data were retrieved through database of Genotypes and Phenotypes (dbGaP) authorization (Accession No. phs000178.v9.p8). Information about TCGA and the investigators and institutions who constitute the TCGA research network can be found at http://cancergenome.nih.gov/. All scripts used for generating figures are available upon request.
