# Supplementary material for: Germline ancestry influences the evolutionary disease course in lung adenocarcinomas
Source: Evol Appl. 2020 Apr 17;13(7):1550–7. doi: 10.1111/eva.12964 (PMC7484830; doi:10.1111/eva.12964)
Supplement: Supplementary file 1 — Fig S1 [file EVA-13-1550-s001.pdf]

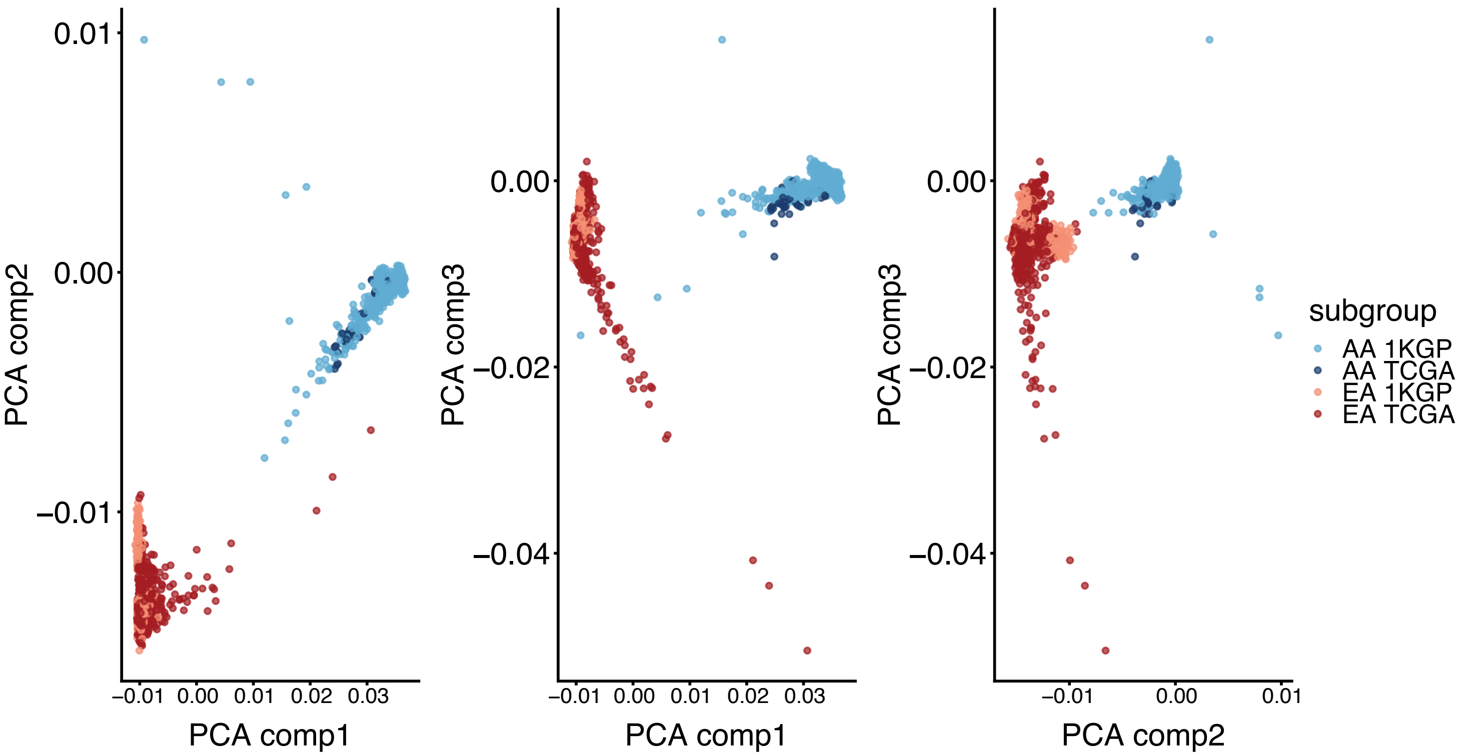

Figure S1. Scatter plot of PCA component 1 against PCA component 2 for 1KGP (light red for EA and light blue for AA) and for TCGA (dark red for EA and dark blue for AA). As the estimated ancestries in TCGA and given ancestries in 1KGP in each group cluster together very well without any overlap to the other group, it is assumable that the admixture analysis is straight.
