# Supplementary material for: Germline ancestry influences the evolutionary disease course in lung adenocarcinomas
Source: Evol Appl. 2020 Apr 17;13(7):1550–7. doi: 10.1111/eva.12964 (PMC7484830; doi:10.1111/eva.12964)
Supplement: Supplementary file 2 — Fig S2 [file EVA-13-1550-s002.pdf]

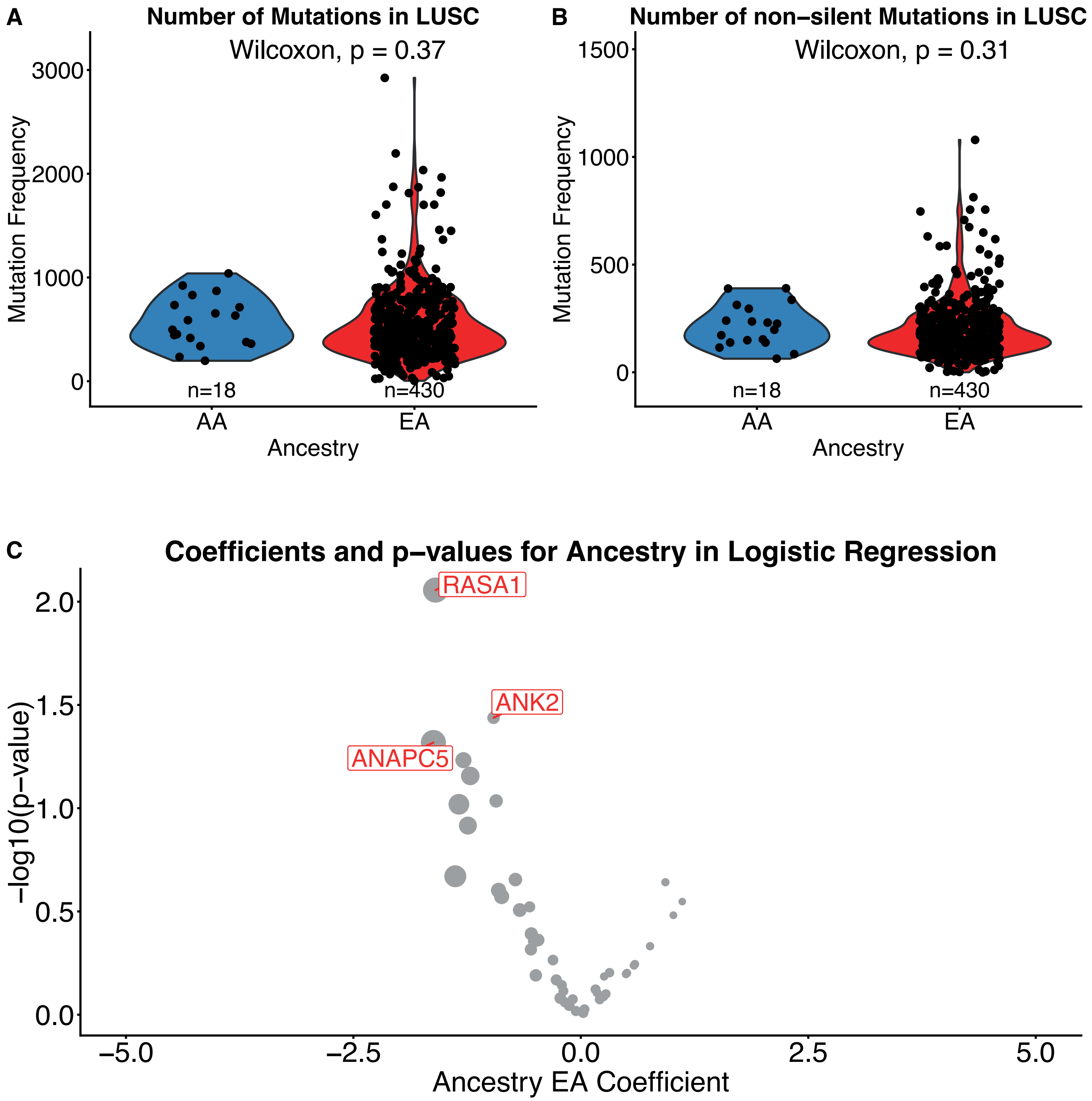

Figure S2. Comparison of mutation burden in LUSC for all mutations (A) and only for non-silent mutations (B). C) Only RASA1 shows significantly different mutation frequencies among EE and EA when applying Fisher's Exact Tests for each driver gene. Besides RASA1, logistic regression revealed ANK2 and ANAPC5 to be differently mutated in the two ancestry groups.
