# Supplementary material for: Germline ancestry influences the evolutionary disease course in lung adenocarcinomas
Source: Evol Appl. 2020 Apr 17;13(7):1550–7. doi: 10.1111/eva.12964 (PMC7484830; doi:10.1111/eva.12964)
Supplement: Supplementary file 3 — Fig S3 [file EVA-13-1550-s003.pdf]

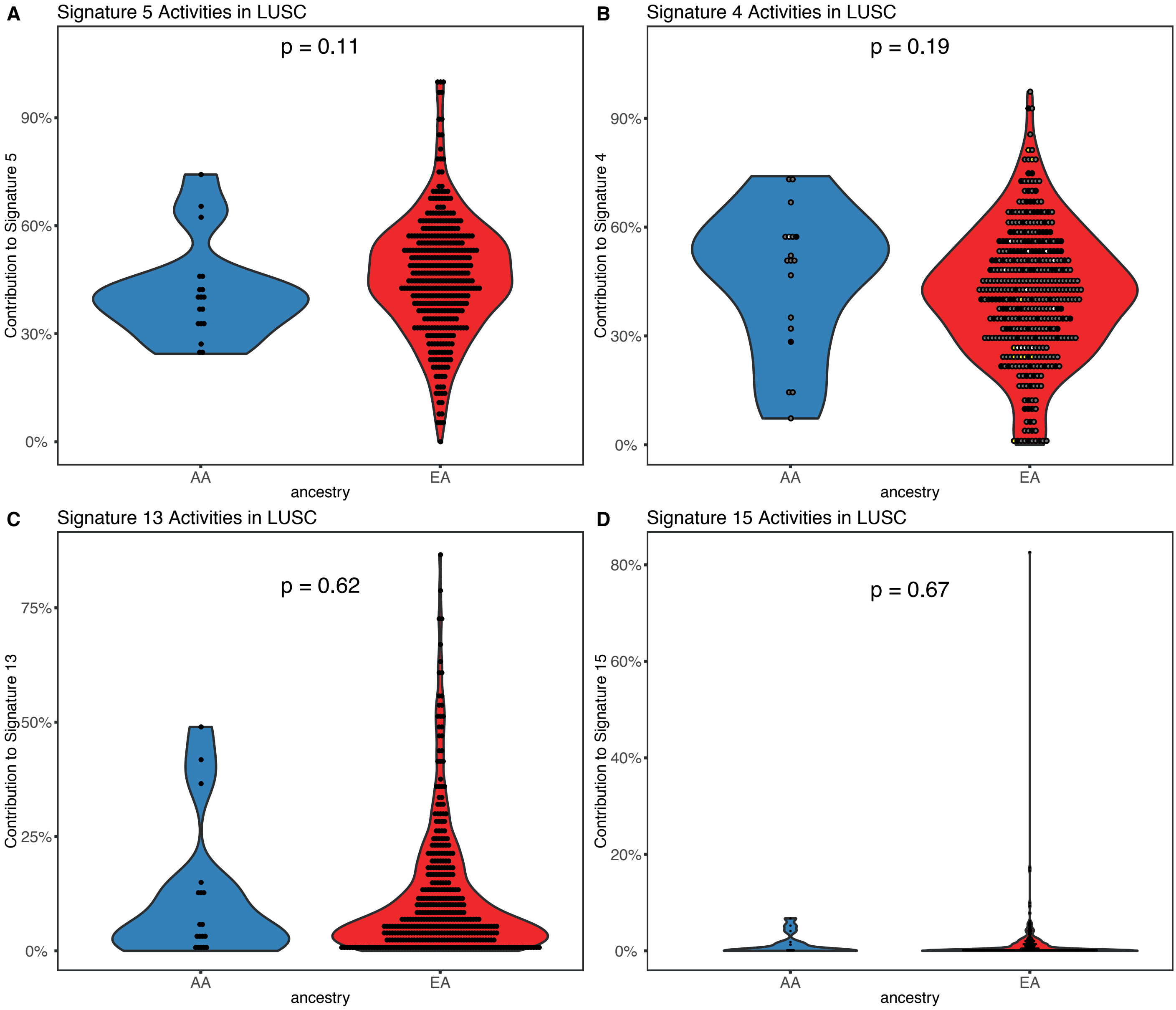

Figure S3 Comparison of mutational signatures between AA and EA identified within LUSC tumours.
