# Supplementary figures and images for: Germline ancestry influences the evolutionary disease course in lung adenocarcinomas
Source: Evol Appl. 2020 Apr 17;13(7):1550–7. doi: 10.1111/eva.12964 (PMC7484830; doi:10.1111/eva.12964)

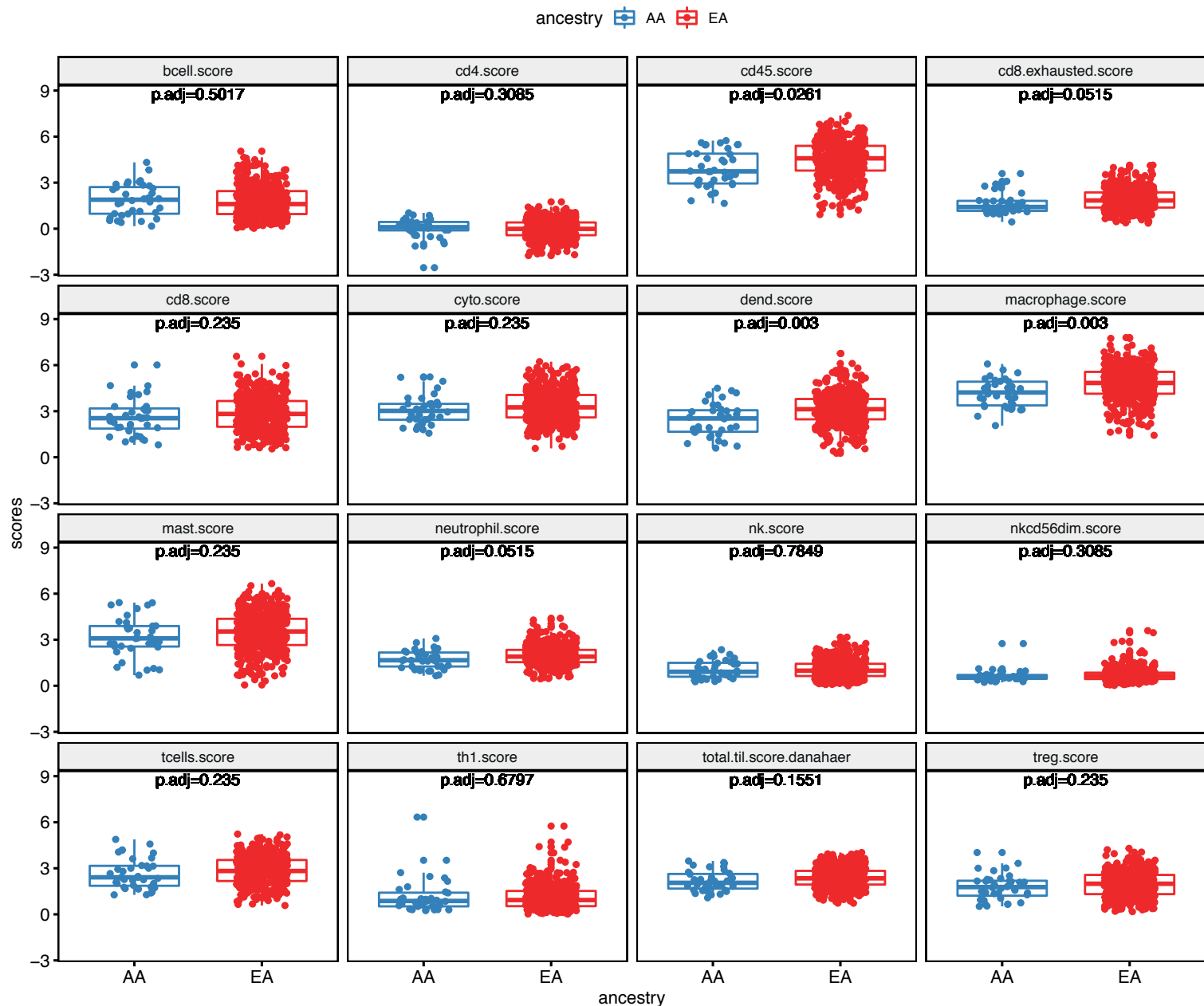

**Figure S4** Comparisons of immune scores between AA and EA LUAD tumours.

Supplement: Supplementary file 4 — Fig S4 [file EVA-13-1550-s004.pdf]
